# An Adaptive Fault-Tolerant Federated Kalman Filter for a Multi-Sensor Integrated Navigation System

**DOI:** 10.3390/s26041360

**Published:** 2026-02-20

**Authors:** Guangle Gao, Guoqing Li, Yingmin Yi, Yongmin Zhong

**Affiliations:** 1 School of Automation and Information Engineering, Xi’an University of Technology, Xi’an 710048, China; gaoguangle@xaut.edu.cn (G.G.); liganma47@gmail.com (G.L.); yiym@xaut.edu.cn (Y.Y.); 2 School of Engineering, RMIT University, Melbourne, VIC 3000, Australia

**Keywords:** integrated navigation, federated Kalman filter, fault tolerance, information factor, hypothesis test

## Abstract

To achieve autonomous and reliable all-weather cross-domain aerospace navigation, this study proposes an adaptive fault-tolerant federated Kalman filter (AFTFKF) for an INS/SRNS/CNS integrated navigation system to enhance system robustness against measurement outliers. First, a noise estimator based on maximum likelihood estimation (MLE) and aided by a sequential probability ratio test (SPRT) is introduced to handle slowly growing outliers. Second, a double residual-based Chi-square test (DCST) information factor is designed to mitigate the impact of inaccurate local state estimation in subsystems under abruptly changed outliers. Finally, the SPRT-MLE-based noise estimator and the DCST-based information factor are integrated into the federated Kalman filter framework to construct the complete AFTFKF. Simulation results demonstrate that the proposed method achieves superior accuracy and strong stability for SINS/SRNS/CNS integrated navigation in the presence of outliers.

## 1. Introduction

Autonomous and reliable all-weather cross-domain aerospace navigation is a fundamental prerequisite to enhance the survivability of aerospace vehicles and ensure the safe and stable completion of transportation missions [1,2]. Currently, the inertial navigation system (INS) serves as the core navigation subsystem for aerospace vehicles [3,4]. INS can provide attitude, velocity, and position information under all-weather conditions, and is characterized by high reliability, strong anti-interference capability, high autonomy, and a high update rate. However, INS suffers from cumulative error growth over time, which limits its ability to maintain safe and reliable navigation performance during long-endurance missions. The global navigation satellite system (GNSS) provides high-accuracy position information based on signals transmitted from artificial satellites and is therefore commonly used to correct INS errors over long-duration operations. Nevertheless, GNSS lacks autonomy and is highly vulnerable to intentional interference and signal blockage [5,6].

The celestial navigation system (CNS) is an autonomous navigation method that determines the position of aerospace vehicles by measuring angular information from celestial bodies. Compared with GNSS, CNS offers the advantages of non-accumulating navigation errors and strong resistance to electromagnetic interference [7,8]. However, traditional CNS does not fully exploit the available information from celestial bodies. When star identification becomes difficult, the measurement information may be insufficient, which can lead to navigation divergence and degraded system performance [9]. The spectral redshift navigation system (SRNS) is a novel celestial navigation technique that estimates vehicle velocity from the spectral redshift of celestial spectra. By integrating SRNS with CNS, both position and velocity information can be obtained autonomously from celestial observations. Consequently, the combined INS/SRNS/CNS integrated navigation system can effectively correct INS errors, fully utilize celestial information, and avoid parameter divergence while preserving system autonomy [9,10,11]. However, Different sensors are heterogeneous and have different accuracy in different environments. Especially in complex environments, measurement outliers can occur, leading to changes in the statistical characteristics of noise. Therefore, finding an appropriate information fusion method to achieve accurate and real-time multi-source navigation information fusion in complex environments is crucial.

## 2. Related Work

The Kalman filter (KF) framework has been the primary information fusion algorithm for linear navigation system integration. To address nonlinear navigation problems, several improved Kalman filtering methods have been developed, including the extended Kalman filter (EKF), unscented Kalman filter (UKF), and cubature Kalman filter (CKF). Among these approaches, CKF demonstrates superior numerical stability compared with UKF [12] and avoids the linearization-induced bias and potential divergence commonly encountered in EKF when dealing with strong nonlinearities [13].

The aforementioned methods are based on centralized filtering architectures. In multi-sensor integrated navigation systems, when certain sensors suffer faults that generate measurement outliers, centralized structures are not well suited for isolating abnormal measurements, resulting in limited fault tolerance and reduced system reliability. To address this issue, Carlson et al. proposed the federated Kalman filter (FKF) framework [14,15]. In this approach, the multi-sensor navigation system is divided into multiple subsystems according to sensor types, and the global state estimate is obtained by fusing the local state estimates of each subsystem. Compared to centralized filtering, where measurement-related parameters are centralized in a single matrix, the measurement information in FKF is separated into lower-dimensional and independent matrices. Thus, this structure enables convenient fault isolation and reduced computational complexity. The earliest FKF was a non-reset architecture, in which the global state of the integrated navigation system and the local states of the subsystems are independent of each other, thereby providing high fault tolerance [16]. However, this structure suffers from relatively low fusion accuracy. Subsequently, FKF schemes based on the information-sharing principle were developed. In these methods, the local state estimates of subsystem filters are replaced by the global state estimate from the master filter, while appropriate information-sharing factors are introduced to mitigate the correlation among subsystem filters, leading to improved filtering accuracy. In practical navigation applications, however, significant performance differences exist among subsystems. Therefore, adaptive selection of information-sharing factors is essential to reflect real-time variations in subsystem states and tracking performance. Kim et al. suggested that the discrepancy between sub-filters and the main filter can serve as a key criterion for determining adaptive information-sharing factors [17], while Wang et al. proposed methods based on innovation covariance matrices [18]. Nevertheless, these adaptive schemes generally suffer from high computational complexity, which limits their practical applicability.

Although FKF provides a convenient framework for fault isolation, it lacks intrinsic capability to detect and identify measurement outliers or sensor faults. Therefore, hypothesis testing methods are commonly integrated with FKF to achieve effective fault detection and isolation, among which the Chi-square test and the sequential probability ratio test (SPRT) are the most representative approaches [19,20,21,22,23]. The Chi-square test is mainly effective in detecting abrupt errors [19,20,21], whereas SPRT is more suitable for identifying slowly growing errors [22,23]. However, FKF typically assumes that measurement noise statistics are known and constant, while in practical applications they are time-varying and uncertain. This mismatch leads to inaccurate test statistics and degrades fault detection performance.

To mitigate the influence of measurement outliers under unknown noise statistics, noise estimation techniques have been incorporated into FKF. In [24,25], maximum likelihood estimation (MLE)-based methods were proposed to estimate measurement noise statistics by maximizing their probability density functions, thereby enhancing system robustness. In [26], covariance matching methods estimate noise statistics by enforcing consistency between innovation covariance and actual measurement covariance. In [27,28], variational Bayesian approaches directly estimate system states by selecting appropriate conjugate prior distributions for measurement noise covariance. These methods can achieve accurate state estimation when noise statistics are constant or slowly varying. However, since these robust Kalman filters rely heavily on historical innovations for noise estimation, they are unable to cope with abruptly changing noise statistics caused by measurement outliers [4]. To address this limitation, Chi-square-test-based noise estimation methods were proposed and integrated with FKF to achieve robust estimation under abruptly changing measurement outliers [29,30]. Although these methods effectively suppress abrupt outliers, they sacrifice estimation accuracy under normal measurement conditions and do not consider slowly growing outliers. In addition, reference [31] constructed an information-sharing factor based on the Chi-square test to reduce the influence of abnormal measurements. Nevertheless, this approach also fails to address slowly growing outliers.

This paper proposes an adaptive fault-tolerant federated Kalman filter (AFTFKF) to improve robustness against measurement outliers in INS/SRNS/CNS integrated navigation systems. A noise estimator based on MLE assisted by SPRT is developed to detect and suppress slowly growing outliers. To address abruptly changing outliers, a double-residual-based Chi-square test (DCST) information factor is introduced to mitigate the influence of inaccurate local state estimation in subsystems. By embedding the SPRT–MLE noise estimator and the DCST information factor into the federated Kalman filter framework, the proposed AFTFKF achieves enhanced fault tolerance and estimation accuracy. Simulation results demonstrate that the proposed method provides superior accuracy and strong stability for SINS/SRNS/CNS integrated navigation under various outlier conditions.

## 3. Model of INS/SRNS/CNS Integrated Navigation System

### 3.1. System State Equation

According to the INS error model, the state equation of INS/SRNS/CNS integrated navigation can be given as

(1) $$X_{k}=FX_{k-1}+W$$

where F denotes the INS system matrix [3], W denotes the system noise matrix, and X denotes the system state vector, specifically expressed as

(2) $$X={\left[\phi_{E}\;\;\;\phi_{N}\;\;\;\phi_{H}\;\;\;\delta v_{E}\;\;\;\delta v_{N}\;\;\;\delta v_{H}\;\;\;\delta L\;\;\;\delta \lambda \;\;\;\delta h\;\;\;\varepsilon_{E}^{b}\;\;\;\varepsilon_{N}^{b}\;\;\;\varepsilon_{H}^{b}\;\;\;\nabla_{E}\;\;\;\nabla_{N}\;\;\;\nabla_{H}\right]}^{T}$$

where $\phi =(\phi_{E},\phi_{N},\phi_{H})$ denotes the platform angle error, $\delta v^{n}=(\delta v_{E},\delta v_{N},\delta v_{H})$ denotes the velocity error in navigation frame (n-frame), $\delta p^{n}=(\delta L,\delta \lambda ,\delta h)$ denotes the position error in n-frame, and $\varepsilon^{b}=(\varepsilon_{E}^{b},\varepsilon_{N}^{b},\varepsilon_{H}^{b})$ and $\nabla =(\nabla_{E},\nabla_{N},\nabla_{H})$ respectively denote the gyro random drift and accelerometer random bias.

### 3.2. Measurement Equation of INS/SRNS Subsystem

Combining the spectral redshift principle with the Doppler frequency shift formula, the relationship between the redshift of celestial spectrum observed from the vehicle and the vehicle velocity can be written as [12]

(3) $$\hat{z}=\frac{c+v_{r}}{\sqrt{c^{2}-{\left|v_{\mathrm{real}}^{i}-v_{c}\right|}^{2}}}-1+\Delta z$$

where $\hat{z}$ denotes the spectral redshift, $v_{\mathrm{real}}^{i}$ denotes the vehicle velocity in the inertial frame (i-frame), $v_{c}$ denotes the velocity vector of the celestial body in the i-frame, which can be obtained by the celestial ephemeris, c is the velocity of light, and $v_{r}$ denotes the radial velocity along the direction from the target vehicle to the observed celestial body, which can be expressed as

(4) $$v_{r}=(v_{\mathrm{real}}^{i}-v_{c})\cdot u$$

where u is the unit direction vector of the observed celestial body in the i-frame, which can be measured by a star sensor.

Thus, it has

(5) $$\hat{z}=\frac{c+v_{\mathrm{real}}^{i}u-v_{c}u}{\sqrt{c^{2}-{\left|v_{\mathrm{real}}^{i}-v_{c}\right|}^{2}}}-1+\Delta z$$

As the velocity calculated by INS in the i-frame can be written as $v_{\mathrm{INS}}^{i}=v_{\mathrm{real}}^{i}+\delta v^{i}$, $\hat{z}$ can be modified as

(6) $$\hat{z}=\frac{c+v_{\mathrm{INS}}^{i}u-v_{c}u-\delta v^{i}u}{\sqrt{c^{2}-{\left|v_{\mathrm{INS}}^{i}-v_{c}-\delta v^{i}\right|}^{2}}}-1+\Delta z$$

with

(7) $$\delta v^{i}=C_{i}^{n}\left[\begin{array}{c} \delta v_{E}\\ \delta v_{N}\\ \delta v_{H} \end{array}\right]=C_{i}^{n}\delta v^{n}$$

where $C_{i}^{n}$ represents the transformation matrix from the n-frame to i-frame.

The measurement equation of the INS/SRNS subsystem can be written as

(8) $$Z_{1}=\hat{z}=h(\delta v^{n})+\Delta z=\frac{c+v_{\mathrm{INS}}^{i}u-v_{c}u-C_{i}^{n}\delta v^{n}u_{i}}{\sqrt{c^{2}-{\left|v_{\mathrm{INS}}^{i}-v_{c}-C_{i}^{n}\delta v^{n}\right|}^{2}}}-1+\Delta z$$

### 3.3. Measurement Equation of INS/CNS Subsystem

The elevation angle measured by CNS can be expressed as [1,29]

(9) $$\sin a_{el}=\sin Dec\sin L_{\mathrm{real}}+\cos Dec\cos L_{\mathrm{real}}\cos\left(t_{\mathrm{GHA}}-\mathrm{Ra}+\lambda_{\mathrm{real}}\right)+\Delta a_{el}$$

where $a_{el}$ is the elevation angle measured by CNS, *Dec* and Ra are the declination and right ascension of the observed celestial body, which can be obtained from the celestial ephemeris, $t_{\mathrm{GHA}}$ is the Greenwich hour angle, $L_{\mathrm{real}}$ and $\lambda_{\mathrm{real}}$ denote the real latitude and longitude of the spacecraft, and $\Delta a_{el}$ is the measurement error.

It is known that

(10) $$\left\{\begin{array}{l} \lambda_{\mathrm{real}}=\lambda_{\mathrm{INS}}-\delta \lambda \\ L_{\mathrm{real}}=L_{\mathrm{INS}}-\delta L \end{array}\right.$$

where $\left(\lambda_{\mathrm{INS}},L_{\mathrm{INS}}\right)$ denotes the longitude and latitude outputs of INS.

Then, the elevation angle measurement equation can be expressed as

(11) $$\begin{array}{l} \sin a_{el}=h(\delta L,\delta \lambda )+\Delta a_{el}\\ =\sin Dec\sin\left(L_{\mathrm{INS}}-\delta L\right)+\cos Dec\cos\left(L_{\mathrm{INS}}-\delta L\right)\times \cos\left(t_{\mathrm{GHA}}-\mathrm{Ra}+\lambda_{\mathrm{INS}}-\delta \lambda \right)+\Delta a_{el} \end{array}$$

In order to prevent the divergence of INS’s altitude channel, a barometric altimeter is involved in CNS. The altitude difference between the barometric altimeter and the INS output is taken as the altimeter measurement, which is given as

(12) $$h_{\mathrm{INS}}-h_{H}=h(\delta h)+\Delta h=\delta h+\Delta h$$

where $h_{\mathrm{INS}}$ and $h_{H}$ denote the altitude outputs from INS and the barometric altimeter, and Δh denotes the measurement error in altitude.

Combining (11) and (12) generates

(13) $$Z_{2}=\left[\begin{array}{c} \sin a_{el}\\ h_{\mathrm{INS}}-h_{H} \end{array}\right]=\left[\begin{array}{c} h(\delta L,\delta \lambda )\\ h(\delta h) \end{array}\right]+\left[\begin{array}{c} \Delta a_{el}\\ \Delta h \end{array}\right]$$

**Figure 1:**
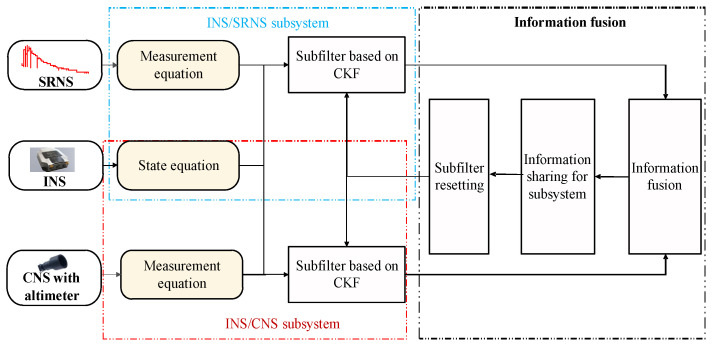
Structure of INS/SRNS/CNS integrated navigation system.

Based on the above equations, the structure of the INS/SRNS/CNS integrated navigation system can be given as Figure 1.

## 4. Federated Kalman Filter with Cubature Kalman Filter for INS/SRNS/CNS Integrated Navigation

In this section, we shall discuss the cubature federated Kalman filter by combining the federated Kalman filter with cubature Kalman filter to achieve the state estimation in the INS/SRNS/CNS integrated navigation system.

### 4.1. Cubature Kalman Filter for Subsystem State Estimation

The cubature Kalman filter (CKF) is mainly used for subsystem state estimation in the INS/SRNS/CNS integrated navigation system. The specific steps of the CKF algorithm are written as follows:

Step 1: State prediction

(14) $$X_{k|k-1}^{t}=F{\hat{X}}_{k-1}^{t}$$

(15) $$P_{k|k-1}^{t}=F_{k}P_{k-1}^{t}F_{k}^{T}+Q_{k}^{t}$$

where $X_{k-1}^{t}$ denotes the state estimation of subsystem *t* at time k − 1, $P_{k-1}^{t}$ denotes the state covariance of t subsystem at time k − 1, $P_{k|k-1}^{t}$ denotes the state covariance of subsystem *t*, and $Q_{k}^{t}$ denotes the process noise covariance.

Step 2: Calculate the gain matrix

(16) $$S_{k|k-1}^{t}{S_{k|k-1}^{t}}^{T}=P_{k|k-1}^{t}$$

(17) $$\varsigma_{i,k|k-1}^{t}=S_{k|k-1}^{t}\xi_{i}+X_{k|k-1}^{t}$$

(18) $$z_{i,k|k-1}^{t}=h(\varsigma_{i,k|k-1}^{t})+r_{k}^{t}$$

(19) $$Z_{t,k|k-1}=\sum_{i=1}^{2n}w_{i}z_{i,k|k-1}^{t}$$

where $r_{k}^{t}$ denotes the mean of measurement noise of subsystem *t*, h(•) denotes the state equation, and $\xi_{i}$ is the line 1 element of $\xi_{n\times 2n}$; the $\xi_{n\times 2n}$ is respectively expressed as

(20) $$\xi_{n\times 2n}={\left[\begin{array}{cccccccc} 1 & 0 & \cdots & 0 & -1 & 0 & \cdots & 0\\ 0 & 1 & & 0 & 0 & -1 & & 0\\ \vdots & \vdots & \ddots & \vdots & \vdots & & \ddots & \\ 0 & 0 & \cdots & 1 & 0 & 0 & \cdots & -1 \end{array}\right]}_{n\times 2n}$$

where *n* is the dimension of state.

Then

(21) $$P_{k|k-1}^{t,zz}=\sum_{i=1}^{2n}w_{i}z_{i,k|k-1}^{t}{z_{i,k|k-1}^{t}}^{T}+Z_{t,k|k-1}{Z_{t,k|k-1}}^{T}+R_{k}^{t}$$

(22) $$P_{k|k-1}^{t,xz}=\sum_{i=1}^{2n}w_{i}\varsigma_{i,k|k-1}^{t}{z_{t,k|k-1}^{t}}^{T}+X_{k|k-1}^{t}{Z_{t,k|k-1}}^{T}$$

where $Z_{t,k|k-1}$ denotes the measurement prediction of subsystem *t*, $P_{k|k-1}^{t,zz}$ denotes the prediction measurement covariance matrix of subsystem *t*, $P_{k|k-1}^{t,xz}$ denotes the cross-covariance matrix of subsystem *t*, and $R_{k}^{t}$ represents the measurement noise covariance of subsystem *t*.

Finally, the gain can be gotten as

(23) $$K_{k}^{t}=P_{k|k-1}^{t,xz}{\left(P_{k|k-1}^{t,zz}\right)}^{-1}$$

Step 3: Update the state with measurement

(24) $${\hat{X}}_{k}^{t}=X_{k|k-1}^{t}+K_{k}^{t}(Z_{t,k}-Z_{t,k|k-1})$$

(25) $${\hat{P}}_{k}^{t}=P_{k|k-1}^{t}+K_{k}^{t}P_{k|k-1}^{zz}K_{k}^{t}$$

where ${\hat{X}}_{k}^{t}$ denotes the state estimation and ${\hat{P}}_{k}^{t}$ denotes the state covariance under subsystem *t*.

### 4.2. Federated Kalman Filter for Information Fusion

After local state estimation of subsystem by CKF, the global state estimation can be obtained by the information fusion through FKF, which is as

(26) $${\hat{P}}_{k}^{g}={\left(\sum {\left({\hat{P}}_{k}^{t}\right)}^{-1}\right)}^{-1}$$

(27) $${\hat{X}}_{k}^{g}={\hat{P}}_{k}^{g}(\sum {\left(P_{k}^{t}\right)}^{-1}{\hat{X}}_{k}^{t})$$

where ${\hat{P}}_{k}^{g}$ is the covariance matrix estimation after global filtering; ${\hat{X}}_{k}^{g}$ is the state estimation after global filtering.

Then the parameter of the sub-filter can be reset as

(28) $${\hat{X}}_{k}^{t}={\hat{X}}_{k}^{g}$$

(29) $$Q_{k}^{t}=\beta_{k}^{t-1}Q_{k}^{g}$$

(30) $${\hat{P}}_{k}^{t}=\beta_{k}^{t-1}{\hat{P}}_{k}^{g}$$

where $\beta_{k}^{t}$ denotes the information sharing factor for subsystem *t*.

## 5. Adaptive Fault-Tolerant Cubature Federated Kalman Filter for INS/CNS/SRNS Integrated Navigation

### 5.1. Noise Estimation Based on MLE and SPRT

To deal with the slow-growing fault, this paper develops a noise estimation algorithm based on MLE and SPRT, which is discussed as below.

Define the innovation

(31) $$\tau_{k}^{t}=Z_{t,k}-Z_{t,k/k-1}$$

Given a sequence of innovations $\left\{\tau_{j}^{t}|j=k-N+1,\cdots ,k\right\}$, the joint probability density can be written as

(32) $$L(\mu_{k}^{t},\Sigma_{k}^{t})=\prod_{j=1}^{N}\frac{1}{2\pi^{m/2}{\left|\Sigma_{k}^{t}\right|}^{1/2}}e^{-\frac{1}{2}{\left(\tau_{k-j+1}^{t}-\mu_{k}^{t}\right)}^{T}{\left(\Sigma_{k}^{t}\right)}^{-1}(\tau_{k-j+1}^{t}-\mu_{k}^{t})}$$

where $\mu_{k}^{t}$ and $\Sigma_{k}^{t}$ respectively denote the expectation, covariance of $\tau_{k}^{t}$, and *N* denotes the size of the moving window.

Applying the logarithmic operation to (32) yields

(33) $$\begin{array}{l} \ln[L(\mu_{k}^{t},\Sigma_{k}^{t})]=\mathrm{In}[\prod_{j=1}^{N}\frac{1}{2\pi^{m/2}{\left|\Sigma_{k}^{t}\right|}^{1/2}}e^{-\frac{1}{2}{\left(\tau_{k-j+1}^{t}-\mu_{k}^{t}\right)}^{T}{\left(\Sigma_{k}^{t}\right)}^{-1}(\tau_{k-j+1}^{t}-\mu_{k}^{t})}]\\ =-\sum_{j=1}^{N}\mathrm{In}[2\pi^{m/2}{\left|\Sigma_{k}\right|}^{1/2}]-\frac{1}{2}\sum_{j=1}^{N}{\left(\tau_{k-j+1}^{t}-\mu_{k}^{t}\right)}^{T}{\left(\Sigma_{k}^{t}\right)}^{-1}(\tau_{k-j+1}^{t}-\mu_{k}^{t})\\ =-\frac{N}{2}\mathrm{In}[2\pi^{m}\left|\Sigma_{k}^{t}\right|]-\frac{1}{2}\sum_{j=1}^{N}{\left(\tau_{k-j+1}^{t}-\mu_{k}^{t}\right)}^{T}{\left(\Sigma_{k}^{t}\right)}^{-1}(\tau_{k-j+1}^{t}-\mu_{k}^{t}) \end{array}$$

According to MLE, the innovation can be estimated as

(34) $${\hat{\mu }}_{k}^{t}=\arg\underset{\mu_{k}^{t}}{\max}\ln[L(\mu_{k}^{t},\Sigma_{k}^{t})]$$

Thus, we have

(35) $$\begin{array}{l} \frac{\partial \ln[L(\mu_{k}^{t},\Sigma_{k}^{t})]}{\partial \mu_{k}^{t}}=\frac{\partial \{N\mathrm{In}[2\pi^{m/2}{\left(\Sigma_{k}^{t}\right)}^{1/2}]+\frac{1}{2}\sum_{j=1}^{N}{\left(\tau_{k-j+1}^{t}-\mu_{k}^{t}\right)}^{T}{\left(\Sigma_{k}^{t}\right)}^{-1}(\tau_{k-j+1}^{t}-\mu_{k}^{t})\}}{\partial \mu_{k}}\\ =\sum_{j=1}^{k}{\left(\Sigma_{k}^{t}\right)}^{-1}(\tau_{k-j+1}^{t}-\mu_{k}^{t})\\ =0 \end{array}$$

Solving (35) yields

(36) $${\hat{\mu }}_{k}^{t}=\frac{1}{N}\sum_{j=1}^{N}\tau_{k-j+1}^{t}$$

In reality, when the estimation of ${\hat{\mu }}_{k}^{t}$ fluctuates slightly near to 0 without slow-growing outliers, the measurement noise will follow the zero-mean Gaussian distribution, and the covariance should still be constant [23].

Based on above, the hypothesis test of SPRT about the mean of innovation can be set as

(37) $$\left\{\begin{array}{l} H_{0}:\;E[\tau_{k}^{t}]=0,E[\tau_{j}^{t}{\tau_{j}^{t}}^{T}]={\hat{\Sigma }}_{k}^{t}\\ H_{1}:\;E[\tau_{k}^{t}]={\hat{\mu }}_{k}^{t},E[(\tau_{j}^{t}-\mu_{k}^{t}){\left(\tau_{j}^{t}-\mu_{k}^{t}\right)}^{T}]={\hat{\Sigma }}_{k}^{t} \end{array}\right.$$

Calculate the likelihood ratio and its logarithm

(38) $$\lambda_{\mu }^{t}(k)=\frac{L(\tau_{k-N+1}^{t},\tau_{2}^{t},\cdots ,\tau_{k}^{t},0,{\hat{\Sigma }}_{k-1}^{t}|H_{1})}{L(\tau_{k-N+1}^{t},\tau_{2}^{t},\cdots ,\tau_{k}^{t},{\hat{\mu }}_{k}^{t},{\hat{\Sigma }}_{k-1}^{t}|H_{0})}=\prod_{j=1}^{N}e^{\frac{{\left(\tau_{k-j+1}^{t}\right)}^{T}{\left({\hat{\Sigma }}_{k-1}^{t}\right)}^{-1}{(}_{k-j+1}^{t})-{\left(\tau_{k-j+1}^{t}-{\hat{\mu }}_{k}^{t}\right)}^{T}{\left({\hat{\Sigma }}_{k-1}^{t}\right)}^{-1}(\tau_{k-j+1}^{t}-{\hat{\mu }}_{k}^{t})}{2}}$$

(39) $$\begin{array}{l} \mathrm{In}[\lambda_{\mu }^{t}(k)]=\sum_{j=1}^{N}\frac{{\left(\tau_{k-j+1}^{t}\right)}^{T}{\left({\hat{\Sigma }}_{k-1}^{t}\right)}^{-1}(\tau_{k-j+1}^{t})}{2}-\sum_{j=1}^{N}\frac{{\left(\tau_{k-j+1}^{t}-{\hat{\mu }}_{k}^{t}\right)}^{T}{\left({\hat{\Sigma }}_{k-1}^{t}\right)}^{-1}(\tau_{k-j+1}^{t}-{\hat{\mu }}_{k}^{t})}{2}\\ =\frac{1}{2}\mathrm{trace}\left(N{{\hat{\mu }}_{k}^{t}}^{T}{\hat{\mu }}_{k}^{t}{{\hat{\Sigma }}_{k-1}^{t}}^{-1}\right)\\ =\frac{N}{2}{{\hat{\mu }}_{k}^{t}}^{T}{{\hat{\Sigma }}_{k-1}^{t}}^{-1}{\hat{\mu }}_{k}^{t} \end{array}$$

According to (39), if the condition $H_{0}$ holds, $\mathrm{In}[\lambda_{\mu }^{t}(k)]$ should be near to zero. Otherwise, $\mathrm{In}[\lambda_{\mu }^{t}(k)]$ will be over a threshold of $T_{\mathrm{SPRT}}\;$. Thus, the mean estimation function of measurement noise can be set as

(40) $${\hat{r}}_{k}=\left\{\begin{array}{l} {\hat{\mu }}_{k}=\frac{1}{N}\sum_{j=1}^{N}\tau_{k-j+1},\;\mathrm{In}[\lambda_{\mu }(k)]>T_{\mathrm{SPRT}}\;\\ 0,\mathrm{In}[\lambda_{\mu }(k)]<T_{\mathrm{SPRT}}\; \end{array}\right.$$

with the threshold $T_{SPRT}\;$ as

(41) $$T_{SPRT}=\mathrm{In}(\frac{1-P_{m}}{P_{f}})$$

where $P_{f}$ represents the false alarm rate and $P_{m}$ the false negative rate.

Meanwhile, the covariance estimation of the innovation can also be obtained under MLE, which is

(42) $${\hat{\Sigma }}_{k}^{t}=\arg\underset{\Sigma_{k}^{t}}{\max}\ln[L({\hat{\mu }}_{k}^{t},\Sigma_{k}^{t})]$$

Then, we have

(43) $$\begin{array}{l} \frac{\partial \ln[L({\hat{\mu }}_{k}^{t},\Sigma_{k}^{t})]}{\partial \Sigma_{k}^{t}}=-\frac{N}{2}\mathrm{trace}[{\left(\Sigma_{k}^{t}\right)}^{-1}]-\frac{\sum_{j=1}^{N}{\left(\tau_{k-j+1}^{t}-{\hat{\mu }}_{k}^{t}\right)}^{T}{\left(\Sigma_{k}^{t}\right)}^{-1}(\tau_{k-j+1}^{t}-{\hat{\mu }}_{k}^{t})\}}{2\partial \Sigma_{k}}\\ =\mathrm{trace}[N\Sigma_{k}^{t-1}-\sum_{j=1}^{N}{\left(\tau_{k-j+1}^{t}-{\hat{\mu }}_{k}^{t}\right)}^{T}(\tau_{k-j+1}^{t}-{\hat{\mu }}_{k}^{t})\Sigma_{k}^{t-2}]\\ =0 \end{array}$$

Solving (43) obtains

(44) $${\hat{\Sigma }}_{k}^{t}=\frac{1}{N}\sum_{j=1}^{N}{\left(\tau_{k-j+1}^{t}-{\hat{r}}_{k}^{t}\right)}^{T}{\left(\tau_{k-j+1}^{t}-{\hat{r}}_{k}^{t}\right)}^{-1}$$

Then, the hypothesis test of SPRT about the covariance of measurement noise can be set as

(45) $$\left\{\begin{array}{l} H_{0}:\;E[(\tau_{j}^{t}-{\hat{\mu }}_{k}^{t}){\left(\tau_{j}^{t}-{\hat{\mu }}_{k}^{t}\right)}^{T}]=\Sigma_{0}^{t}\\ H_{1}:\;E[(\tau_{j}^{t}-{\hat{\mu }}_{k}^{t}){\left(\tau_{j}^{t}-{\hat{\mu }}_{k}^{t}\right)}^{T}]={\hat{\Sigma }}_{k}^{t} \end{array}\right.$$

where

(46) $$\Sigma_{0}=\sum_{i=1}^{2n}w_{i}z_{i,k|k-1}^{t}{z_{i,k|k-1}^{t}}^{T}+Z_{t,k|k-1}{Z_{t,k|k-1}}^{T}+R_{0}$$

where $R_{0}$ is the initial measurement covariance.

Then, calculate the likelihood ratio and its logarithm

(47) $$\lambda_{\Sigma }^{t}(k)=\frac{L(\tau_{k-N+1}^{t},\tau_{2}^{t},\cdots ,\tau_{k}^{t},{\hat{\mu }}_{k}^{t},\Sigma_{0}^{t}|H_{1})}{L(\tau_{k-N+1}^{t},\tau_{2}^{t},\cdots ,\tau_{k}^{t},{\hat{\mu }}_{k}^{t},{\hat{\Sigma }}_{k}^{t}|H_{0})}=\prod_{j=1}^{N}e^{\frac{{\left(\tau_{k-j+1}^{t}-\mu_{k}^{t}\right)}^{T}{\left(\Sigma_{0}^{t}\right)}^{-1}(\tau_{k-j+1}^{t}-\mu_{k}^{t})-{\left(\tau_{k-j+1}^{t}-\mu_{k}^{t}\right)}^{T}{\left({\hat{\Sigma }}_{k}^{t}\right)}^{-1}(\tau_{k-j+1}^{t}-\mu_{k}^{t})}{2}}$$

(48) $$\mathrm{In}[\lambda_{\Sigma }^{t}(k)]=\sum_{j=1}^{N}\frac{{\left(\tau_{k-j+1}^{t}-\mu_{k}^{t}\right)}^{T}{\left(\Sigma_{0}^{t}\right)}^{-1}(\tau_{k-j+1}^{t}-\mu_{k}^{t})}{2}-\sum_{j=1}^{N}\frac{{\left(\tau_{k-j+1}^{t}-\mu_{k}^{t}\right)}^{T}{\left({\hat{\Sigma }}_{k}^{t}\right)}^{-1}(\tau_{k-j+1}^{t}-\mu_{k}^{t})}{2}$$

Similar to the hypothesis test of SPRT about the mean of innovation, if the condition $H_{0}$ holds, $\mathrm{In}[\lambda_{\Sigma }^{t}(k)]$ should be near to zero. Otherwise, $\mathrm{In}[\lambda_{\Sigma }^{t}(k)]$ will be over a threshold of $T_{\mathrm{SPRT}}\;$. The mean estimation function of measurement noise can be set as

(49) $${\hat{R}}_{k}^{t}=\left\{\begin{array}{l} \frac{1}{N}\sum_{j=1}^{N}{\left(\tau_{k-j+1}^{t}-{\hat{r}}_{k}^{t}\right)}^{T}{\left(\tau_{k-j+1}^{t}-{\hat{r}}_{k}^{t}\right)}^{-1}\\ -\sum_{i=1}^{2n}w_{i}z_{i,k|k-1}^{t}{z_{i,k|k-1}^{t}}^{T}-Z_{t,k|k-1}{Z_{t,k|k-1}}^{T},\;\;\;\;\;\;\;\mathrm{In}[\lambda_{\Sigma }^{t}(k)]>T_{\mathrm{SPRT}}\;\\ R_{0}^{t},\mathrm{In}[\lambda_{\Sigma }^{t}(k)]<T_{\mathrm{SPRT}}\; \end{array}\right.$$

As shown in (40) and (49), the proposed noise estimator introducing SPRT can determine whether changes in ***r*** and ***R*** are generated by a slow-growing outlier or merely caused by fluctuations from limited innovation samples. This prevents overestimation by MLE and better distinguishes slow-growing outliers from fluctuations due to limited innovation samples, thereby providing a more accurate estimation of the statistical characteristics of noise in the presence of slow-growing outliers.

### 5.2. DCST-Based Information Factor

Under complex faults leading to slow-growing and abruptly changed outliers, MLE- and SPRT-based noise estimation cannot deal with abruptly changed outliers due to the involvement of historical information. To deal with this problem, a DCST-based information factor is introduced in the MLE- and SPRT-based noise estimation.

The residuals from the local state estimation and global estimation can be written as

(50) $$\gamma_{k}^{t}={\hat{X}}_{k}^{t}-X_{k/k-1}^{t}$$

(51) $$\gamma_{g,k}^{t}={\hat{X}}_{k}^{g}-{\hat{X}}_{k}^{t}$$

Then, the covariances of the residuals can be written as

(52) $${\hat{\Sigma }}_{k}^{X,t}=\gamma_{k}^{t}\gamma_{k}^{tT},{\hat{\Sigma }}_{g,k}^{X,t}=\gamma_{g,k}^{t}\gamma_{g,k}^{tT}$$

Further, the hypothesis test based on the residuals from the local state estimation can set as

(53) $$Test\;1:\left\{\begin{array}{l} H_{0}:\;E[\gamma_{k}^{t}\gamma_{k}^{tT}]={\hat{\Sigma }}_{k}^{X,t}\\ H_{1}:\;E[\gamma_{k}^{t}\gamma_{k}^{tT}]\neq {\hat{\Sigma }}_{k}^{X,t} \end{array}\right.$$

According to the Chi square test, the judge index based on the residuals from the local state estimation can be expressed as

(54) $$Test\;1:\;\lambda_{C}^{t}(k)=\gamma_{k}^{t}{\left(P_{k/k-1}^{t}\;\right)}^{-1}\gamma_{k}^{tT}$$

where $\lambda_{C}^{t}(k)∼\chi {\left(n\right)}^{2}$.

Similarly, the hypothesis test based on the residuals from the global state estimation can be set as

(55) $$Test\;2:\left\{\begin{array}{l} H_{0}:E[\gamma_{g,k}^{t}\gamma_{g,k}^{tT}]={\hat{\Sigma }}_{g,k}^{X,t}\\ H_{1}:\;E[\gamma_{g,k}^{t}\gamma_{g,k}^{tT}]\neq {\hat{\Sigma }}_{g,k}^{X,t} \end{array}\right.$$

Then judge index based on the residuals from the global estimation can be expressed as

(56) $$Test\;2:\;\lambda_{g,C}^{t}(k)=\gamma_{g,k}^{t\;\;\;\;T}{\left({\hat{P}}_{g,k}\right)}^{-1}\gamma_{g,k}^{t}$$

where $\lambda_{g,C}^{t}(k)∼\chi {\left(n\right)}^{2}$.

Combining (54) and (56), the judgement function of DCST can be obtained as

(57) $$\left\{\begin{array}{l} Case\;1,\lambda_{C}^{t}(k)\leq T_{C},\lambda_{g,C}^{t}(k)\leq T_{C}\;\\ Case\;2,\lambda_{C}^{t}(k)\leq T_{C},\lambda_{g,C}^{t}(k)>T_{C}\\ Case\;3,\lambda_{C}^{t}(k)>T_{C},\lambda_{g,C}^{t}(k)\leq T_{C}\\ Case\;4,\lambda_{C}^{t}(k)>T_{C},\lambda_{g,C}^{t}(k)>T_{C} \end{array}\right.$$

where $T_{C}\;$ is the threshold, which can be found by the chi-square distribution.

Since the INS error model is accurate in short time, $X_{k/k-1}^{t}$ will be accurate, leading to the following four cases:

Case 1: From (57), it can be seen that $\lambda_{C}^{t}(k)$ will be small and under the threshold. This means ${\hat{X}}_{k}^{t}$ is accurate and the measurement does not involve abruptly changed outlier. Meanwhile, $\lambda_{g,C}^{t}(k)$ is also under the threshold, which means the difference between ${\hat{X}}_{k}^{t}$ and ${\hat{X}}_{k}^{g}$ is small. Thus, this accurate ${\hat{X}}_{k}^{t}$ will have a high contribution to estimate ${\hat{X}}_{k}^{g}$, leading to accurate global fusion.

Case 2: $\lambda_{C}^{t}(k)$ is smaller than threshold $T_{C}\;$, which means ${\hat{X}}_{k}^{t}$ is accurate, while $\lambda_{g,C}^{t}(k)$ is greater than the threshold which means that this accurate ${\hat{X}}_{k}^{t}$ has a low contribution to the estimation of ${\hat{X}}_{k}^{g}$.

Case 3: $\lambda_{C}^{t}(k)$ is greater than the threshold which means that ${\hat{X}}_{k}^{t}$ is inaccurate and measurement involves abruptly changed outliers, while $\lambda_{g,C}^{t}(k)$ is smaller than the threshold which means that this inaccurate ${\hat{X}}_{k}^{t}$ has a high contribution to the estimation of ${\hat{X}}_{k}^{g}$, leading to a negative influence on global fusion.

Case 4: $\lambda_{C}^{t}(k)$ is greater than the threshold which means that ${\hat{X}}_{k}^{t}$ is inaccurate and measurement involves abruptly changed outliers. However, since $\lambda_{g,C}^{t}(k)$ is also greater than the threshold, the contribution of the inaccurate ${\hat{X}}_{k}^{t}$ to the estimation of ${\hat{X}}_{k}^{g}$ is small and negligible.

It can be observed that the subsystem in Case 1 exhibits no obvious abruptly changing outliers and contributes significantly to the global state estimation, indicating that the corresponding state covariance in (25) is appropriate. In Case 2, although no apparent abruptly changing outliers are present, the subsystem contributes less to the global estimation. Therefore, the corresponding state covariance in (25) should be adjusted during fusion to enhance its positive contribution. In Case 3, the subsystem contains significant outliers, leading to the inaccurate estimation of measurement noise statistics while still contributing strongly to the global estimation. Consequently, the corresponding state covariance in (25) should be reduced to suppress its negative influence. In contrast, Case 4 shows that although significant outliers are present, the subsystem contribution is low; hence, the corresponding state covariance estimation remains reasonable and requires no further adjustment.

Based on above, the information fusion factor for state covariance estimation of a subsystem before global fusion can be set as

(58) $$\alpha_{k}^{t}=\left\{\begin{array}{l} \lambda_{C}^{t}(k)/\lambda_{g,C}^{t}(k),\;\mathrm{for}\;\mathrm{Case}\;2\;\mathrm{and}\;\mathrm{Case}\;3\\ 1,\;\mathrm{for}\;\mathrm{Case}\;1\;\mathrm{and}\;\mathrm{Case}\;4 \end{array}\right.$$

Then, (26) and (27) can be updated as

(59) $${\hat{P}}_{k}^{*g}={\left(\sum {\left(\alpha_{k}^{t}{\hat{P}}_{k}^{t}\right)}^{-1}\right)}^{-1}$$

(60) $${\hat{X}}_{k}^{*g}={\hat{P}}_{k}^{g}(\sum {\left(\alpha_{k}^{t}{\hat{P}}_{k}^{t}\right)}^{-1}{\hat{X}}_{k}^{t})$$

Through (59) and (60), the state covariance estimation can be adjusted to the suitable value by $\alpha_{k}^{t}$, leading to accurate estimation under abruptly changed outliers.

Further, the local estimation has been reset by the global state estimation based on (28), and thus we have

(61) $${\hat{X}}_{k}^{t}={\hat{X}}_{k}^{*g}$$

In order to reflect the variance and estimate the statistical characteristics of the state after resetting, the information sharing factor is set based on the judge index of Test2, which is as

(62) $$\beta_{k}^{t}=\lambda_{g,C}^{t}(k)/\sum \lambda_{g,C}^{i}(k)$$

As shown in (62), ${\hat{X}}_{k}^{t}$ with a smaller contribution will be reset to ${\hat{X}}_{k}^{*g}$, leading to a larger $\beta_{k}^{t}$ to the corresponding subsystem to decrease the state covariance estimation to match the more accurate ${\hat{X}}_{k}^{*g}$. Otherwise, ${\hat{X}}_{k}^{t}$, which makes a higher contribution, is already matched to ${\hat{X}}_{k}^{*g}$, thus only a relatively small $\beta_{k}^{t}$ is needed.

### 5.3. AFTFKF in Multi-Sensor Integration

**Figure 2:**
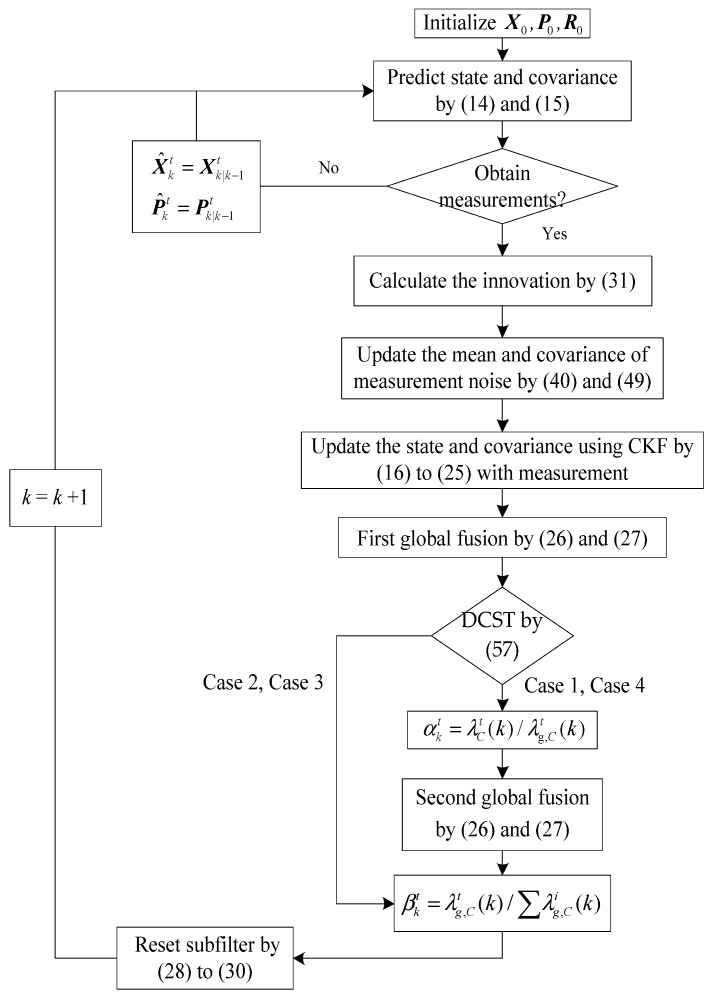
Procedure of AFTFKF.

Consequently, based on the above derivations, the procedure of AFTFKF for INS/SRNS/CNS is as presented in Figure 2 and the following main steps:

Step 1. Initialize the matrix of $X_{0}$, $P_{0}$, $R_{0}$ and Q.

Step 2. Compute the innovations as (31), then based on SPRT-aided MLE, estimate the ${\hat{r}}_{k}^{t}$ and ${\hat{R}}_{k}^{t}$ of the subsystem by (40) and (49).

Step 3. Estimate ${\hat{X}}_{k}^{t}$ and ${\hat{P}}_{k}^{t}$ by CKF from (14) to (25).

Step 4. Choose the (26) and (27) to achieve the first-time information fusion.

Step 5. Calculate the information fusion factor $\alpha_{k}^{t}$ by (58).

Step 6. Choose the (59) and (60) to achieve the second-time information fusion.

Step 7. Calculate the information sharing factor $\beta_{k}^{t}$ by (62) and reset the subsystem parameters by (61), (29) and (30).

Step 8. Repeat steps 2 to 6 until the navigation ends.

## 6. Simulation and Analysis

Simulations were conducted to comprehensively evaluate the performance of AFTFKF for an INS/SRNS/CNS integrated navigation system. AFTFKF is compared to FKF, Chi-square test-based FKF(CSTFKF) [29] and adaptive information sharing factor aided FKF(AISFKF) [31].

**Figure 3:**
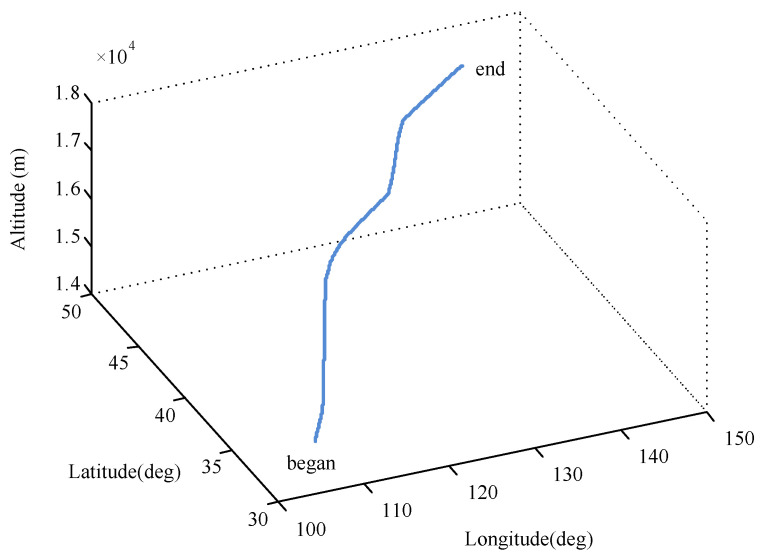
Flight trajectory.

The flight trajectory was designed following the actual flight (see Figure 3). The simulation parameters are shown in Table 1 and the simulation time was set to 1000 s. The filtering period was 1 s and the window size *N* was set to 50 s.

The initial noise covariances of SRNS and CNS measurement are set as

(63) $$R_{0}^{z}\left[\begin{array}{ccc} 10^{-8} & 0 & 0\\ 0 & 10^{-8} & 0\\ 0 & 0 & 10^{-8} \end{array}\right],R_{0}^{\mathrm{el}}=\left[\begin{array}{ccc} 5{}^{\circ} & 0 & 0\\ 0 & 5{}^{\circ} & 0\\ 0 & 0 & 50\;m \end{array}\right]$$

where $R_{0}^{z}$ represents the initial noise covariance of SRNS measurement and $R_{0}^{\mathrm{el}}$ the initial noise covariance of CNS measurement.

The absolute velocity error and absolute position error are calculated as

(64) $$\Delta V=\sqrt{\Delta v_{E}^{2}+\Delta v_{N}^{2}+\Delta v_{H}^{2}}$$

(65) $$\Delta P=\sqrt{\Delta L^{2}+\Delta \lambda^{2}+\Delta H^{2}}$$

The root mean square error (RMSE) and mean absolute error (MAE) are defined as

(66) $$\mathrm{RMSE}(\Delta x)=\sqrt{\frac{1}{T}\sum_{k=1}^{T}{\left[\Delta x(k)\right]}^{2}}$$

(67) $$\mathrm{MAE}(\Delta x)=\frac{1}{T}\sum_{k=1}^{T}\Delta x(k)$$

where Δx is ΔV or ΔP, and *T* denotes the number of Monte Carlo runs, which was set to 50.

### 6.1. Accuracy Analysis Under Condition with Slow-Growing Outlier in SRNS Measurement

To verify the performance of the proposed AFTFKF in terms of slow-growing outliers, the SRNS measurements involved slow-growing outliers and were set as

(68) $$\Delta z∼\left\{\begin{array}{l} N(0,R_{0}^{z}),0<k\leq 300\\ N(0.06(k-{300)*10}^{-8},R_{0}^{z}),300<k\leq 400\\ N(0,R_{0}^{z}),400<k \end{array}\right.$$

**Figure 4:**
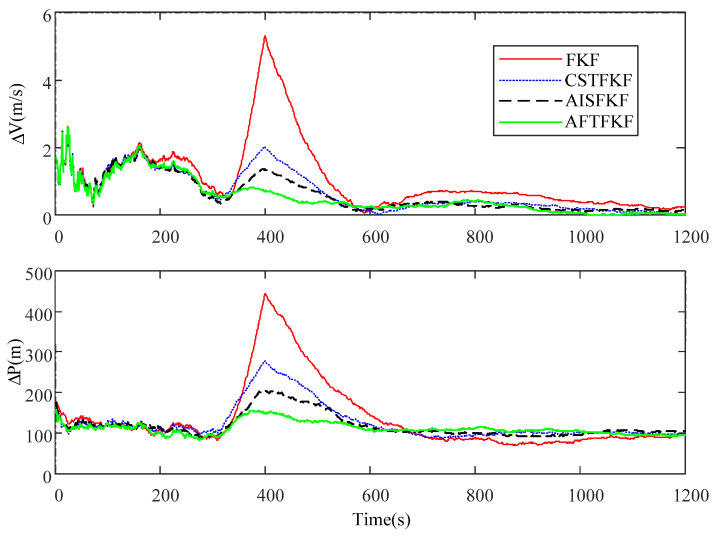
Absolute velocity and position errors of FKF, CSTFKF, AISFKF and AFTFKF under SRNS measurement with outliers.

Figure 4 shows the absolute errors in velocity and position by FKF, CSTFKF, AISFKF and AFTFKF under SRNS measurement with slow-growing outliers. The corresponding MAEs are listed in Table 2.

As shown in Figure 4, before the occurrence of slow-growing outliers and in the absence of changes in the SRNS measurement noise statistics, the absolute position and velocity errors of FKF, CSTFKF, AISFKF and AFTFKF are comparable. In the period (300 s, 400 s), due to the lack of a noise estimator and adaptive information factor, FKF exhibits the largest velocity and position errors, with MAEs of 1.79 m/s and 221.79 m, respectively. By incorporating noise estimators, CSTFKF and AISFKF achieve smaller absolute errors than FKF, with velocity and position MAEs of 1.15 m/s and 169.19 m for CSTFKF, and 0.91 m/s and 150.80 m for AISFKF. However, both methods show limited capability in handling slow-growing outliers. In contrast, by employing the SPRT-aided MLE noise estimator, AFTFKF effectively handles slow-growing outliers, leading to the smallest velocity and position MAEs of 0.51 m/s and 107.78 m, respectively.

**Figure 5:**
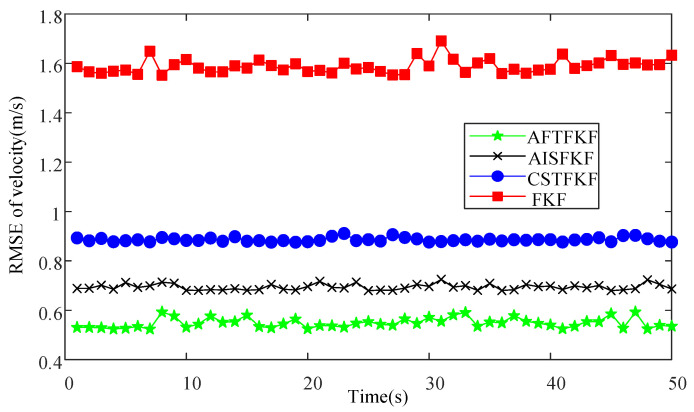
Velocity RMSEs of FKF, CSTFKF, AISFKF and AFTFKF under SRNS measurement with outliers.

**Figure 6:**
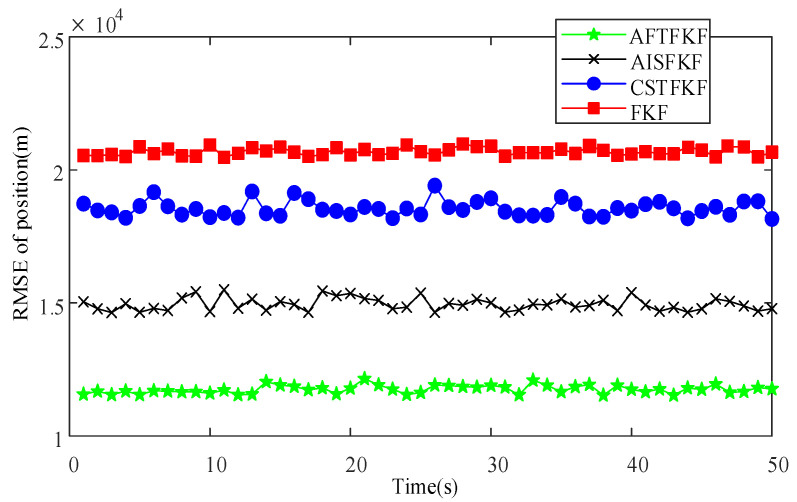
Position RMSEs of FKF, CSTFKF, AISFKF and AFTFKF under SRNS measurement with outliers.

The RMSEs of velocity and position by FKF, CSTFKF, AISFKF and AFTFKF are shown in Figure 5 and Figure 6. The conventional FKF exhibits the poorest estimation performance, with velocity and position RMSEs in the ranges (1.5 m/s, 1.7 m/s) and (2.0 × 10^4^ m, 2.1 × 10^4^ m), respectively, indicating a high sensitivity to outliers and limited robustness. By incorporating robust filtering mechanisms, CSTFKF and AISFKF achieve noticeable improvements in estimation accuracy. Specifically, CSTFKF reduces the velocity RMSE to (0.85 m/s, 0.95 m/s) and the position RMSE to (1.8 × 10^4^ m, 1.9 × 10^4^ m), while AISFKF further lowers them to (0.70 m/s, 0.85 m/s) and (1.5 × 10^4^ m, 1.6 × 10^4^ m), respectively. Moreover, the proposed AFTFKF consistently attains the lowest RMSEs, maintaining velocity RMSEs within (0.55 m/s, 0.60 m/s) and position RMSEs within (1.1 × 10^4^ m, 1.2 ×10^4^ m).

### 6.2. Accuracy Analysis Under Condition with Abruptly Changed Outlier in CNS Measurement

To verify the performance of the proposed AFTFKF in terms of the abruptly changed measurement outliers, the CNS measurements were contaminated by abruptly changed measurement outliers in the following configuration

(69) $$\left(\Delta a_{el},\Delta h\right)∼\left\{\begin{array}{l} N(0,R_{0}^{el}),0<k\leq 300\\ N(0,4R_{el,0})+N(2,3R_{0}^{el}),500<k\leq 600\\ N(0,R_{0}^{el}),600<k \end{array}\right.$$

**Figure 7:**
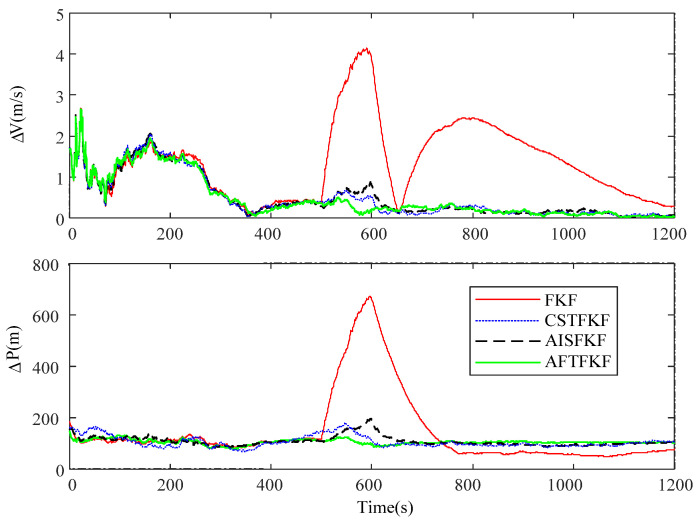
Absolute errors of velocity and position by FKF, CSTFKF, AISFKF and AFTFKF under CNS measurement with outliers.

Figure 7 illustrates the absolute velocity and position errors obtained using FKF, CSTFKF, AISFKF, and AFTFKF under CNS measurements with abruptly changing outliers. The corresponding MAEs for velocity and position are summarized in Table 3.

During the initial time interval without outliers in CNS measurements, the estimation differences in FKF, CSTFKF, AISFKF and AFTFKF are negligible. However, during the period (500 s, 600 s) with the abruptly changed outliers in CNS measurements, FKF exhibits large position and velocity errors, with MAEs of 3.27 m/s and 464.91 m, respectively. Both CSTFKF and AISFKF significantly improve FKF, yielding velocity and position MAEs of 0.59 m/s and 159.72 m for CSTFKF, and 0.63 m/s and 162.51 m for AISFKF. In contrast, AFTFKF achieves further performance gains, reducing the velocity and position MAEs to 0.44 m/s and 125.52 m, respectively.

**Figure 8:**
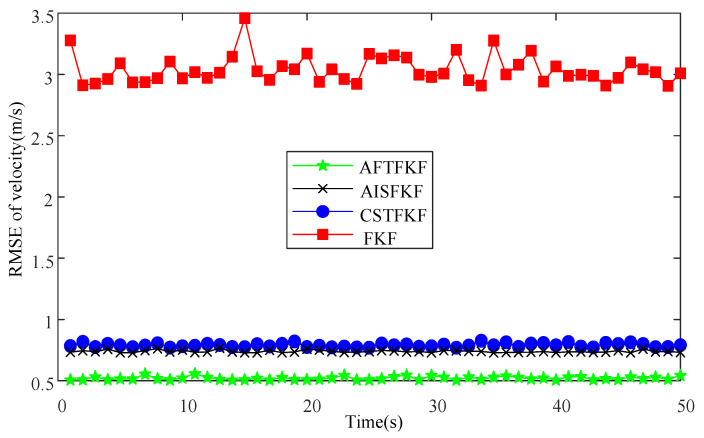
Velocity RMSEs of FKF, CSTFKF, AISFKF and AFTFKF under CNS measurements with outliers.

**Figure 9:**
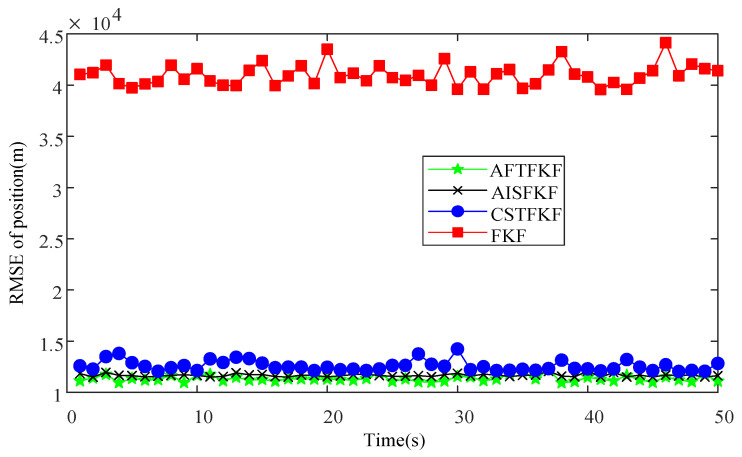
Position RMSEs of FKF, CSTFKF, AISFKF and AFTFKF under CNS measurements with outliers.

Figure 8 and Figure 9 present the velocity and position RMSEs obtained using FKF, CSTFKF, AISFKF and AFTFKF under CNS measurements with outliers. It can be observed that the conventional FKF is highly sensitive to outliers. Its velocity and position RMSEs remain in a relatively high range (2.9 m/s, 3.5 m/s) and (3.9 × 10^4^, 4.4 × 10^4^ m), respectively. CSTFKF and AISFKF achieve noticeable reductions in estimation error. Specifically, CSTFKF reduces the velocity RMSEs to (0.77 m/s, 0.83 m/s) and the position RMSEs to (1.2 × 10^4^, 1.5 × 10^4^ m), while AISFKF lowers the velocity RMSEs to (0.73 m/s, 0.77 m/s) and the position RMSEs to (1.1 × 10^4^, 1.3 ×10^4^ m). Further, the proposed AFTFKF consistently attains the lowest RMSEs across all simulation runs, demonstrating superior robustness and estimation accuracy. Its velocity RMSEs are stably maintained within (0.50 m/s, 0.56 m/s), while the position RMSEs are further reduced to (1.0 × 10^4^ m, 1.2 ×10^4^ m), accompanied by the smallest variability among all compared methods.

### 6.3. Computational Time Analysis

**Figure 10:**
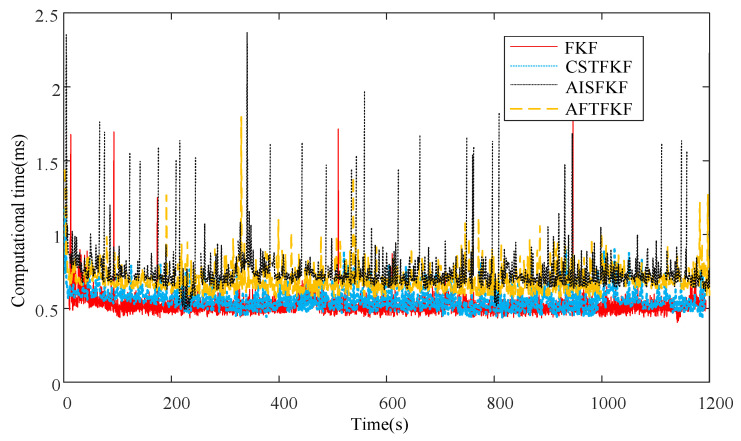
Computational time comparison.

Figure 10 illustrates the computational times of FKF, CSTFKF, AISFKF and AFTFKF using a PC with CPU 13th Gen Intel(R) Core(TM) i5-13500H with 2.60 GHz. Table 4 lists the corresponding mean computational times.

Due to the lack of information factor calculation and noise estimation, FKF has the smallest computational load, with a mean computational time of 0.526 ms. By incorporating CST-based noise estimation, CSTFKF incurs a slightly higher computational cost, with a mean computational time of 0.554 ms. In addition, due to the inclusion of both information factor calculation and noise estimation, AISFKF and AFTFKF require additional computation, resulting in mean computational times of 0.663 ms and 0.627 ms, respectively. Nevertheless, all compared methods, including the proposed AFTFKF, satisfy the real-time performance requirement of 0.07 s per filter period [32]. Thus, the proposed AFTFKF can be used for information fusion for a navigation system which has a high real-time requirement.

In summary, the above simulation results demonstrate that the proposed AFTFKF not only effectively handles both abruptly changed and slow-growing measurement outliers, but also maintains excellent real-time navigation performance compared to the other methods.

## 7. Conclusions

This paper proposes an adaptive fault-tolerant federated Kalman filter (AFTFKF) to address the challenge of measurement outliers in INS/SRNS/CNS integrated navigation systems. The core contributions of this work are twofold. First, a noise estimator based on maximum likelihood estimation (MLE) and aided by the sequential probability ratio test (SPRT) is developed to effectively detect and compensate for slow-growing measurement outliers. Second, a double-residual-based Chi-square test (DCST) information factor is designed to autonomously adjust the information weights within the federated filter, thereby mitigating the adverse effects of inaccurate local state estimates caused by abruptly changing measurement outliers. The integration of these two mechanisms into the federated Kalman filter framework yields a comprehensive and adaptive fault-tolerant solution. The performance of the proposed AFTFKF has been validated through simulations. The results demonstrate that, in the presence of both slow-growing and abruptly changing outliers, the proposed method significantly enhances the estimation accuracy and robustness of SINS/SRNS/CNS integrated navigation compared with conventional approaches, while exhibiting strong stability and maintaining reliable navigation performance.

Future work will focus on constructing adaptive information factors that can simultaneously reflect state errors and the measurement outlier, thereby achieving FKF algorithms that can be applied to a wider range of and more complex navigation scenarios, and further enhancing the reliability of INS/SRNS/CNS integrated navigation system.

## Figures and Tables

**Table 1:** Sensor parameters in simulations.

Gyro parameters	Constant drift White noise Sampling frequency	0.1°/h $0.1{}^{\circ}/\sqrt{h}$ 50 Hz
Accelerometer parameters	Zero bias White noise Sampling frequency	0.1 mg $0.1\;\mathrm{mg}/\sqrt{s}$ 50 Hz
CNS	Elevation angle accuracy Sampling frequency	10° 1 Hz
Barometric altimeter	Altitude error Sampling frequency	50 m 1 Hz
Spectrometer	Redshift accuracy Sampling frequency	10^−8^ 1 Hz

**Table 2:** Position and velocity MAEs of FKF, CSTFKF, AISFKF and AFTFKF under SRNS measurements with outliers.

Methods	Parameter	MAE	
		The Period of (300 s, 400 s)	Other Time
FKF	Velocity Position	1.79 m/s 221.79 m	0.81 m/s 105.28 m
CSTFKF	Velocity Position	1.15 m/s 169.19 m	0.68 m/s 121.47 m
AISFKF	Velocity Position	0.91 m/s 150.80 m	0.62 m/s 114.71 m
AFTFKF	Velocity Position	0.51 m/s 107.78 m	0.40 m/s 106.67 m

**Table 3:** Position and velocity MAEs of FKF, CSTFKF, AISFKF and AFTFKF under CNS measurements with outliers.

Methods	Parameter	MAE	
		The Period of (500 s, 600 s)	Other Time
FKF	Velocity Position	3.27 m/s 464.91 m	1.25 m/s 115.82 m
CSTFKF	Velocity Position	0.59 m/s 159.72 m	0.68 m/s 108.04 m
AISFKF	Velocity Position	0.63 m/s 162.51 m	0.51 m/s 104.13 m
AFTFKF	Velocity Position	0.44 m/s 125.53 m	0.49 m/s 103.60 m

**Table 4:** The mean computational times.

Methods	Mean Computational Time
FKF	0.526 ms
CSTFKF	0.554 ms
AISFKF	0.627 ms
AFTFKF	0.663 ms

## Data Availability

The original contributions presented in this study are included in the article. Further inquiries can be directed to the corresponding author.
